# Metastasis of Lung Adenocarcinoma to the Gingiva: A Rare Case Report

**Published:** 2015-05

**Authors:** M. Rajini Kanth, A. Ravi Prakash, Y. Raghavendra Reddy, J.K. Sonia Bai, M. Ravindra Babu

**Affiliations:** 1 Department of Oral Pathology, G. Pulla Reddy Dental College and Hospital, Andhra Pradesh, India;; 2 Department of Oral Surgery, G. Pulla Reddy Dental College and Hospital, Andhra Pradesh, India;; 3 Onsurgeon, Viswa Bharathi Cancer Hospital, R.T. NAGAR, Penchikalapadu, Kurnool, India

**Keywords:** Lung carcinoma, Mastasis, Mouth mucosa, Gingival

## Abstract

Metastatic tumors account for 1% of all oral malignancies. Metastasis to jaw bones is common, particularly in the mandible, rare in the oral soft tissues, and account for only 0.1% of oral malignancies. The majority of metastatic cases (70%) reported in the literature have primary tumors located in the lung, breast, kidney, and colon. Metastasis is a biological complex process that involves detachment from the surrounding cells, regulation of cell motility, invasion, survival, proliferation, and evasion of the immune system. Clinical presentation of metastatic tumors is variable, which may create diagnostic dilemma or may lead to erroneous diagnosis. Metastatic tumors clinically mimic as dental infections. Metastasis to the oral soft tissue from lung cancer, especially gingiva is a rare condition. Metastasis to the gingiva can affect the oral function, speech, and nutrition. Most of the cases in the literature reported that lesion presented in oral soft tissues before the diagnosis of primary tumors. Here we report a case of 62-year-old male patient with metastasis from lung to the gingiva, where the metastasis was detected before primary tumor.

## Introduction

Distant metastasis of malignant tumors to the oral soft tissues is rare and account for 0.1% of all oral malignancies.^1^^,^^2^ Nearly 90% of metastatic tumors occur in jaw bones especially premolar-molar region of the mandible. Metastasis to the soft tissues mostly involves gingiva (54%) followed by alveolar mucosa (50%) and tongue (30%).^3^^,^^4^ In 25% of cases, oral metastatic tumors are found to be the first sign of metastatic spread and in 23% of cases; they are an indication of unidentified primary metastatic tumor of distant site.^1^ Metastasis to the oral cavity is often the first manifestation of lung cancer.^5^ Common sources of metastasis to the oral region are from the breast, lung, and kidney.^6^ Approximately 70% of patients who were dying of cancer had evidence of metastatic disease.^6^

Biopsy is required for the diagnosis of metastatic tumors in the oral region, but when oral metastasis itself is the first presentation, immunohistochemical stains are necessary to characterize the primary tumor.^2^ The case reported here was presented with only an ulcerated lesion mimicking pyogenic granuloma and finally turned out to be lung cancer as the primary tumor metastasizing to the gingiva. As a result, this article emphasizes on detailed dento-alveolar examination and early diagnosis to find the primary focus of metastatic tumor.

## Case Report

An apparently healthy 62-year-old male patient presented with a swelling in the right lower teeth region of the jaw for a month. The patient’s personal and medical history was non-contributory. On extraoral examination, a well-defined swelling of size 3×3 cm was noted on the right side of the mandible extending from the angle of the mouth to 3 cm in front of the tragus of the ear anteroposteriorly. No other pathologic finding was noticed during the physical examination.

**Figure 1:**
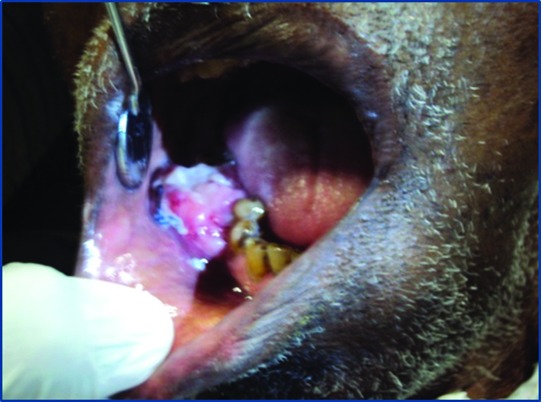
Intra-oral view showing an ulcerated swelling at the recent extraction site.

On intraoral examination, a polypoid exophytic lesion with an ulcerated growth of size 4×5 cm was noted in the right mandibular premolar-molar region (figure 1). The lesion was asymptomatic, polypoid in nature with firmness in consistency. The colour of the lesion was normal with inflammed borders. The lesion extended from the mesial side of 44 to the distal side of 47. The history revealed recent extraction of tooth 46 followed by swelling during the previous month. Provisional diagnosis was given as pyogenic granuloma.

**Figure 2:**
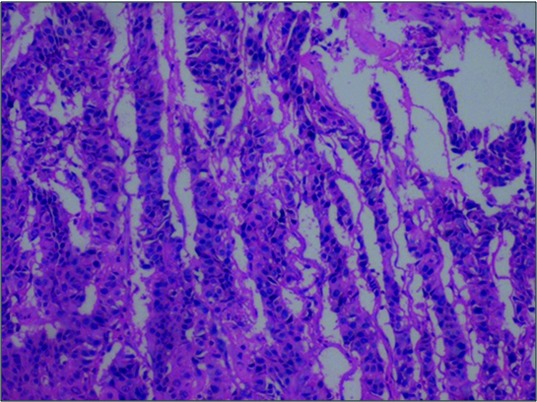
Histological section of the biopsied lesion, demonstrating round to polygonal cells separated by fibrous septae (H&E staining 4× view).

Excisional biopsy was done and histopathological examination showed round to polygonal cells arranged around the alveolar spaces separated by fibrous septae (figure 2) with areas of ossification. Few cells showed pleomorphism with hyperchromatic nuclei.

The tumoral configuration was compatible with poorly differentiated adenocarcinoma, but this morphology is not commonly seen in tumors of oral cavity, including salivary gland tumors, which are known for their diverse morphological and histological features. For this reason, it was thought that the tumor was primarily metastatic. This high-grade adenocarcinoma was thought to be organized from the lung, thyroid, or gastro-intestinal system.

**Figure 3:**
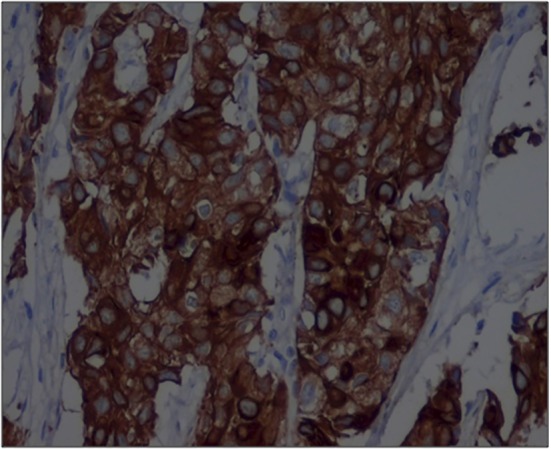
Immunohistochemical profile of tumor cells showing positive staining for cytokeratin 7 (Cytokeratin 7 ×100).

The tissues were sent for immunohistochemistry (IHC) for confirmatory diagnosis. IHC showed poorly differentiated adenocarcinoma expressing cytokeratin 7 (figure 3) and thyroid transcription factor 1, whereas cytokeratin 20 and smooth muscle actin were negative. The pattern suggested metastatic adenocarcinoma from the lung.

**Figure 4:**
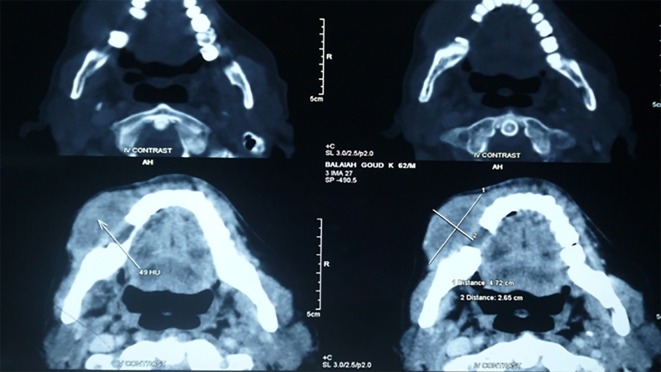
CT scan showing extensive lytic lesion involving the ramus and condyle with destruction of inferior border of mandible.

The patient was advised for a total body scan. The total body computed tomographic (CT) scan with contrast-enhancing medium revealed a large 4.7×2.6 cm heterogeneously enhancing growth at the right lower alveolus with focal bony erosion and large 6.8×5.9 cm heterogeneous mass in the anterior segment of the left upper lobe with focal infiltration of mediastinum abutting the pulmonary trunk. The CT scan from the head region showed an extensive lytic lesion involving the ramus and condyle with the destruction of the inferior border of the mandible (figure 4). PA X-ray chest radiograph revealed a large mass lesion present in the left suprahilar region with an elevated left dome of diaphragm and fibro-calcific lesion in the right apex. No other metastases were detected and his bone scan was negative. After diagnosing the lesion, the case was referred to a higher oncology center for further evaluation and treatment. The patient started radiotherapy followed by chemotherapy. Radiation with a total dosage of 2700 cGy in 10 fractions was given for ten days. Patient response to radiotherapy was good. It was followed by chemotherapy with review for every 3 weeks. The treatment is ongoing and the patient is under follow up.

## Discussion

Metastasis of malignant tumors to the oral mucosa accounts for only 0.1% of all oral malignancies.^4^ In 2008, Hirshberg et al. reviewed 673 cases of oral metastasis, out of which 112 cases were metastasized from the lung, of which 58 were noted in the jawbones and 54 in the oral mucosa.^7^ The mean age of occurrence was 54 years (range of 9-88 years) with slight male predilection.^8^ Primary tumors have been detected in most of the patients before the metastatic spread to the oral cavity.^9^ In our case, the patient was asymptomatic and was not aware of the lung cancer. Metastasis to the soft tissues appear as dental or periodontal infection and they resemble reactive lesions or benign tumors such as pyogenic granuloma, epulis, peripheral giant cell granuloma, and odontogenic infection.^10^ The site of spread of metastasis to the oral cavity from distant organs is determined by the presence of teeth.^2^ In the dentulous patients, metastasis occurs in the gingiva and in edentulous patients, it occurs in between the tongue and alveolar mucosa.^2^

Pathogenesis of oral metastasis is unclear, but thought to be a multistage process in which cells detach themselves from the primary tumor and transported by lymphatic or blood vessels. In oral soft tissues, chronically inflamed mucosa, especially gingiva, has rich capillary network, which can trap the malignant cells and cause metastasis.^11^ Another mechanism is the role of Batson’s plexus, a prevertebral venous network without valves, that authorizes the retrograde direction of crossing of tumor cells towards the face from lungs.^5^ Passage of tumor cells in the gingival tissue can be facilitated by the greatest permeability of vessels and the presence of adhesive molecules.^12^ Primary site of the lesion varies according to the colonization in the oral cavity. The lung is the most common primary site in males, affecting oral mucosa (31.3%) and the breast in females, affecting soft tissues with 24.3%, respectively.^7^

Oral metastatic tumors are late complications and grow rapidly causing pain, difficulty in chewing, dysphagia, disfigurement, and bleeding.^2^ In case of gingival metastasis, clinical distinction becomes difficult between benign and malignant lesion.^13^ It can be suspected for malignancy when there is fast growth, tendency for bleeding, mechanical disorders due to tumor development, an ulcerated lesion, and patient’s clinical condition.^5^ In such cases, there is a possibility for metastasis and biopsy is thus mandatory.^3^ In some cases, metastasis has been detected after a recent dental extraction as seen in our case.^14^ It is a great challenge to the surgeon for appropriate management of the metastatic tumors when retrograde diagnosis is often required.^1^ Immunohistochemistry (IHC) has to be done for most of the cases after histopathological examination for further confirmatory diagnosis. We have confirmed the diagnosis as metastatic lung adenocarcinoma by IHC.

Most of the metastatic tumors in the oral cavity reported in the literature, till date, are with a known primary tumor, which have been already diagnosed, whereas in our case, the patient was asymptomatic and unaware of lung cancer. Tumor in the extracted socket was the initial clinical finding at the time of presentation, which led to the diagnosis of lung cancer. This article emphasizes on detailed dento alveolar examination and early diagnosis for finding the primary focus of metastatic tumor, which will help in a better prognosis of patients. 

Treatment of gingival metastasis depends upon its presentation. It may present as either an initial lesion or late complication during the treatment of lung cancer. Oral metastatic tumors are commonly associated with metastasis to the multiple organs and they are associated with poor outcome and difficult to palliate.^2^ Systemic chemotherapy, radiotherapy or surgical excision of the lesion under local anaesthesia is the treatment modalities.^10^ Average survival rate for lung cancer metastasis is 4-month to 1-year with a maximum survival rate of five years.^15^

## Conclusion

As the oral soft tissue metastasis is rare, diagnosis of metastatic lesions in the oral region is challenging, both to the clinician and to the pathologist. Dental practitioners should suspect that gingival masses, which mimic benign lesions sometimes, represent the underlying malignant tumors. Thorough examination of the patient and biopsy of the existing lesion should be carried out for definitive diagnosis. Early detection of gingival metastasis and appropriate treatment are necessary to improve the quality of life. 
